# First-principles study of the structural, optoelectronic and thermophysical properties of the π-SnSe for thermoelectric applications

**DOI:** 10.3762/bjnano.12.82

**Published:** 2021-10-05

**Authors:** Muhammad Atif Sattar, Najwa Al Bouzieh, Maamar Benkraouda, Noureddine Amrane

**Affiliations:** 1 Physics Department, College of Science, United Arab Emirates University (UAEU), 15551, Al Ain, UAE; 2 National Water and Energy Center (NWEC), United Arab Emirates University (UAEU), 15551, Al Ain, UAE

**Keywords:** density functional theory (DFT), electronic properties, lattice thermal conductivity, optical properties, thermodynamic properties, thermoelectric properties, tin selenide (SnSe)

## Abstract

Tin selenide (SnSe) has thermoelectric (TE) and photovoltaic (PV) applications due to its exceptional advantages, such as the remarkable figure of merit (*ZT* ≈ 2.6 at 923 K) and excellent optoelectronic properties. In addition, SnSe is nontoxic, inexpensive, and relatively abundant. These aspects make SnSe of great practical importance for the next generation of thermoelectric devices. Here, we report structural, optoelectronic, thermodynamic, and thermoelectric properties of the recently experimentally identified binary phase of tin monoselenide (π-SnSe) by using the density functional theory (DFT). Our DFT calculations reveal that π-SnSe features an optical bandgap of 1.41 eV and has an exceptionally large lattice constant (12.2 Å, *P*2_1_3). We report several thermodynamic, optical, and thermoelectric properties of this π-SnSe phase for the first time. Our finding shows that the π-SnSe alloy is exceptionally promising for the next generation of photovoltaic and thermoelectric devices at room and high temperatures.

## Introduction

Thermoelectric (TE) materials convert the direct and reversible heat energy into electrical energy and offer a probable solution and feasible route for power generation alternatives as well as refrigeration through the accumulation of waste heat [1–3] or solar energy [4]. The main obstacle is, however, to create effective, stable, as well as affordable TE materials. The efficiency of TE materials and devices is quantified by the dimensionless figure of merit (*ZT*) which is represented by

[1] $$ZT=\left(\frac{S^{2}\sigma }{\kappa_{\text{tot}}}\right)T=\left(\frac{S^{2}\sigma }{\kappa_{\text{e}}+\kappa_{\text{l}}}\right)T,$$

where *S*, σ, κ_tot_, κ_e_, κ_l_, and *T* represent the Seebeck coefficient, total electrical conductivity, thermal conductivity, electronic thermal conductivity, lattice thermal conductivity, and absolute temperature, respectively [5]. The bottleneck that limits the extensive use of TE materials is their relatively low value of *ZT*. The close interdependency of the three entities *S*, σ, and κ_tot_ offer challenges in the formulation of strategies to improve the *ZT* values of the TE materials, particularly by utilizing affordable and Earth-abundant materials [1,6], and this is the task that may revolutionize the field of solid-state thermal energy conversion [7]. A higher value of *ZT* needs a large value of the power factor (PF = *S*^2^σ) which is connected with the electrical transport [8–9] and the lowest value of κ_tot_ [10]. To date, a considerable amount of research has been performed to enhance the *ZT* value. For instance, by lowering the value of the lattice thermal conductivity (through all-scale hierarchical architecture [2,11–12] as well as through nanostructuring [13–15]), retaining the hole mobility [16–17], and by improving the value of the Seebeck coefficient (by tuning the band structure [18] along with a large conduction (valence) band convergence [19–20], electron energy barrier filtering [21], and quantum confinement effects [22]). Many of these methods focus on reducing the lattice thermal conductivity and try to preserve a high power factor (PF).

On the other hand, a high figure of merit can be obtained in pristine TE materials that have intrinsically minimal thermal conductivity, which might occur through a few properties, such as a complex crystal lattice [14], large molecular mass [23], and charge density wave distortions [24]. In recent years, numerous thermoelectric alloys have shown an outstanding thermoelectric efficiency with *ZT* values greater than two by reasonable thermoelectric models [25–26]. For instance, AgSbTe_2_ when doped with Se shows *ZT* ≈ 2.1 at 573 K [27], GeTe (*ZT* ≈ 2.4 at 600 K by doping with Pb and Bi) [28], PbTe (*ZT* ≈ 2.51 at 823 K by doping with Na, Eu, and Sn) [29], and SnSe crystals (*ZT* ≈ 2.8 at 773 K by doping with Br) [30].

Among the tin-based binary chalcogenide SnX alloys (X = S, Se, and Te), the tin selenide (SnSe) compound belongs to the IV–VI semiconductor family and is the most studied TE material [1,5,31–34]. Tin selenide consists of economical, Earth-abundant, and nontoxic elements and has potential applications in the next generation of electronic and photonic systems [35–36]. The orthorhombic α-SnSe, an indirect bandgap (0.9 eV) semiconductor, has been an immense research topic in the TE field since the highest *ZT* value of ≈2.6 at 923 K was reported in the p-type single crystal along the *b* axis [1]. The n-type Bi-doped SnSe single crystal also gives a high *ZT* value of 2.2 (along the *b* axis) at 773 K [37]. Motivated by these prominent TE performances, which were due to ultra-low thermal conductivity along with modest electrical transport properties, SnSe-based TE alloys have drawn considerable attention lately [38]. In recent years, numerous research groups from all over the globe tried to enhance TE efficiency through alloying [38], doping (metal or hole) [39–43], and texturing [44]. Despite demonstrating superior TE properties, SnSe still has limitations for practical applications mostly as a result of rigid controlled lattice growth conditions, poor elastic properties, and high cost for mass production [45].

Recently, a new cubic crystal structure of tin monoselenide (π-SnSe) (analogous to π-SnS [46]) has been characterized within binary phase by using X-ray diffraction and transmission electron microscopy. As SnS and SnSe share a lot in common, both chemically and structurally, an experimental study verified that the π-SnS prototype exists in the form of π-SnSe. The phase stability of π-SnSe is also confirmed by Golan et al. [47]. By using density functional theory (DFT) calculations along with an experimental characterization they concluded that π-SnSe is energetically close to the stable α-SnSe phase. A working solar cell made by a cubic SnS crystalline structure is also reported [48] with optical bandgaps in the range of 1.6 to 1.8 eV [49]. Therefore, it is natural to explore the optoelectronic properties of π-SnSe for applications in solar energy as well.

As, this new cubic π-SnSe phase has a large lattice constant (*a*_0_ = 11.88 Å) and belongs to the class of non-centrosymmetric crystals, promising and intriguing valuable properties might occur in this alloy. In this paper, we have theoretically explored the physical properties of this new binary phase of cubic π-SnSe alloy. Although one study has experimentally demonstrated the mechanical stability of the π-SnSe phase [47], to the best of our knowledge, optical, thermodynamic, and thermoelectric properties of this newly cubic π-SnSe phase have not been yet investigated from first-principles theory. This study may improve our knowledge about the new binary phase of π-SnSe which can aid to TE and photonic applications of this intriguing π-SnSe alloy.

## Methods

To explore the structure, optoelectronic, and thermophysical properties of the π-SnSe alloy, we have used first-principles calculations based on the full-potential linearized augmented plane-wave (FP-LAPW) method, implemented in the WIEN2k code [50–51]. A preliminary crystal lattice is initially made by using the details presented in [46] and then atomic coordinates along with the volume of the π-SnSe system are allowed to fully relax. We used the PBEsol exchange-correlation functional [52] and the PAW projections were carried out within the reciprocal space. For more accurate electronic structures (e.g., bandgap), we used the computationally inexpensive modified Becke–Johnson (mBJ) meta-GGA exchange-correlation functional over the optimized structure of π-SnSe generated with PBEsol. For a variety of semiconductors and insulators, the mBJ potential calculates the bandgap in a good agreement with experimental data [53]. To ensure a good energy convergence and to avoid charge leakage, we selected the cutoff basis set of *R*_min_ × *K*_max_ = 8, where *R*_min_ is the minimum radius of the elements and *K*_max_ is the value of the main wave vector of the reciprocal lattice for the plane-wave expansion. We used periodic boundary conditions and employ a Γ-centered *k*-point mesh of 4 × 4 × 4 for the Brillouin zone sampling. A denser *k*-mesh of 27 × 27 × 27 (924 *k*-points) is selected to more accurately calculate the optical and thermoelectric parameters. The thermodynamic variables have been computed through the use of the quasi-harmonic Debye model carried out within the Gibbs2 code [54] by considering lattice vibrations. To calculate the thermoelectric properties, we used the Boltzmann transport theory employed in the BoltzTrap2 [55] code by utilizing the rigid band estimation under a constant relaxation time.

## Results and Discussion

### Structural properties

**Figure 1:**
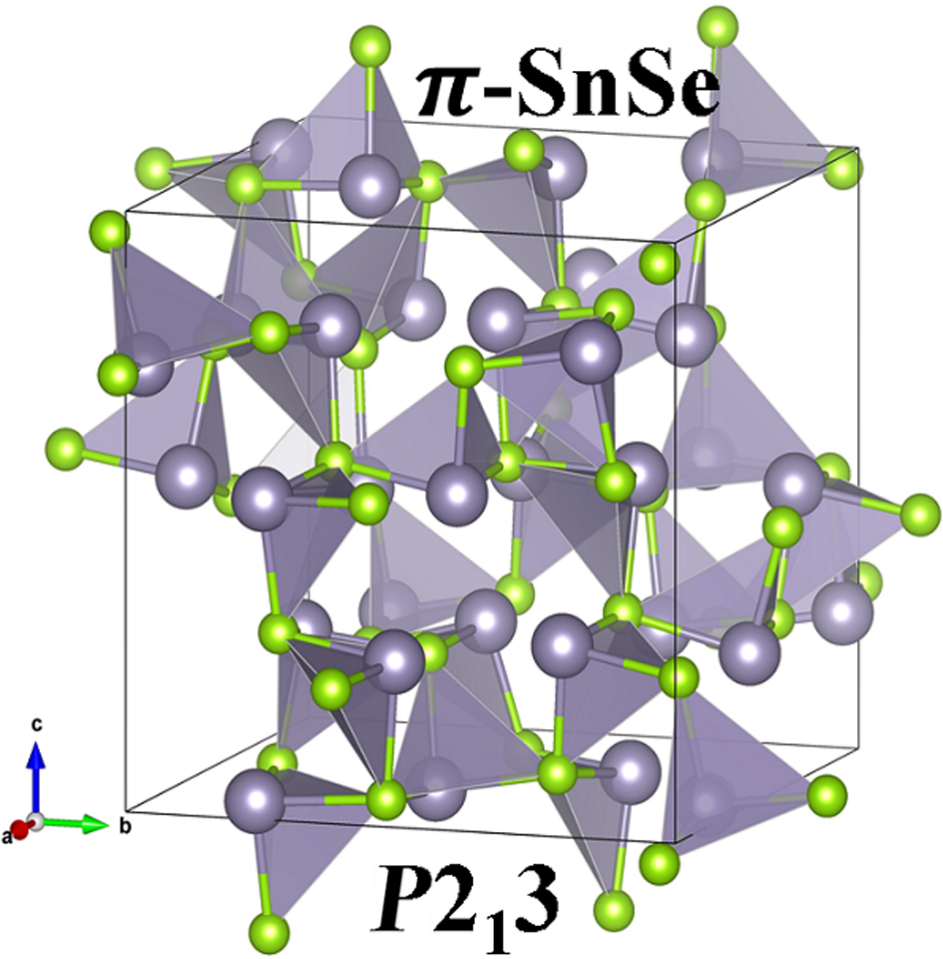
The unit cell of the π-SnSe phase with 64 atoms (Sn = 32, Se = 32) which has a space group *P*2_1_3 (no. 198) and a lattice constant of 12.20 Å. Grey and green colors represents the Sn and Se atoms, respectively. Each Sn atoms sits on the apex of the trigonal pyramidal surrounded by three Se atoms at the trigonal base with a Sn–Se bond distance of 2.6 Å.

We have prepared the initial structure of the π-SnSe by using experimentally determined atomic coordinates of the π-SnS system by replacing the S ion with Se ions due to the equivalent cubic analog of the π-SnS system [46]. Regarding the crystal structure of π-SnSe, Golan and his team, in 2016, first designated the space group of *P*2_1_3 (no. 198) of the 64-atom unit cell with an exceptionally large lattice parameter of 11.9702 Å according to X-ray diffraction using the Rietveld method [47]. The unit cell of π-SnSe is presented in Figure 1.

**Figure 2:**
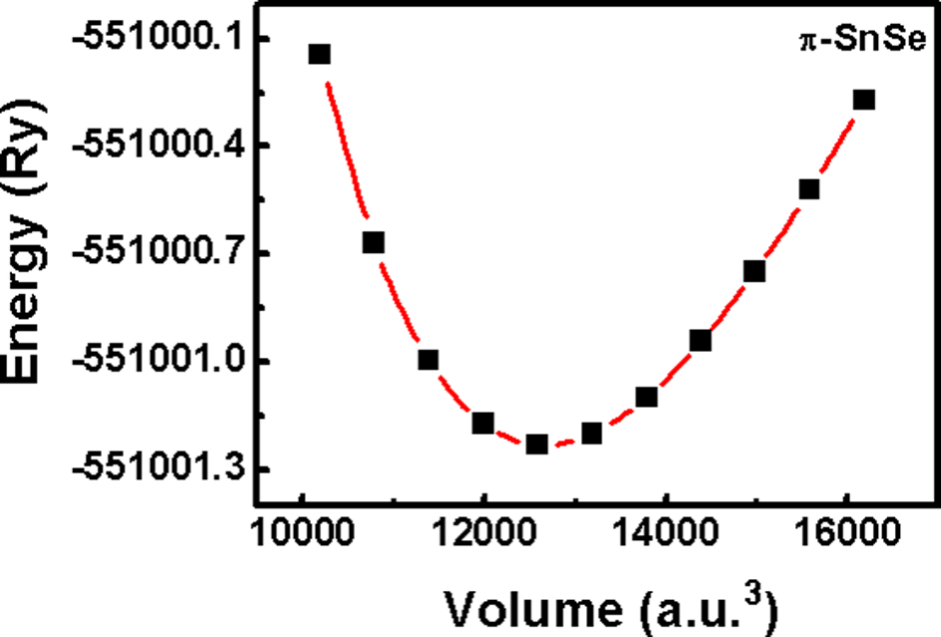
Volume–energy optimization curve of the π-SnSe fitted to the Murnaghan’s equation of state. The relaxed lattice constant is *ab* = 12.108 Å and the optimized volume is *V*_o_ = 12658.093 a.u.^3^.

There are 64 atoms (Sn = 32, Se = 32) in the primitive unit cell of the π-SnSe system. In the cubic π-SnSe crystal structure, every Sn is located at the apex of the trigonal pyramidal connected through three Se atoms at the trigonal base with an Sn–Se bond distance of 2.6 Å. The polyhedrons are linked to produce an unusual corner-sharing system. We started from experimentally reported atomic positions and performed structural optimizations by varying the volume until the minimum energy was reached. The obtained data was fitted into the Murnaghan’s equation of state that can be expressed as:

[2] E(V)=Eo+9VoBo16{[(VoV)23−1]3B′o+[(VoV)23−1]2[6−4[VoV]23]},

where *E*(*V*) and *E*_o_ are the total energies at the deformed (*V*) and reference (*V*_o_) volumes, respectively, and *B*_o_ and 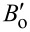 represent the bulk modulus and its first-order pressure derivative, respectively [9,56–57]. The volume–energy optimization curve of the π-SnSe is shown in Figure 2.

The computed ground-state structural parameters are presented in Table 1. For the π-SnSe system, our DFT optimized lattice parameter of 12.108 Å obtained from the PBEsol functional matches well with the experimentally reported lattice parameter (11.9702 Å), as PBE GGA gives a slightly higher value of 12.284 Å [47].

**Table 1:** Lattice parameters for the π-SnSe phase of a 64-atom unit cell. The values of bulk modulus (*B* in GPa) and the derivative of bulk modulus (*B'*), volume (*V* in a.u.^3^), and bond length of Sn–Se (Å) are given below.

Parameter	Present DFT study	Other DFT	Experimental

α (Å)	12.108	12.284^a^	11.9702^a^
*V* (a.u.)	12658.093	—	—
*B*	51.8804	—	—
*B'*	4.4238	—	—
*E* (Ry)	−551001.2308	—	—
Sn–Se ( Å)	2.6	—	—

^a^Reference [47].

### Electronic properties

**Figure 3:**
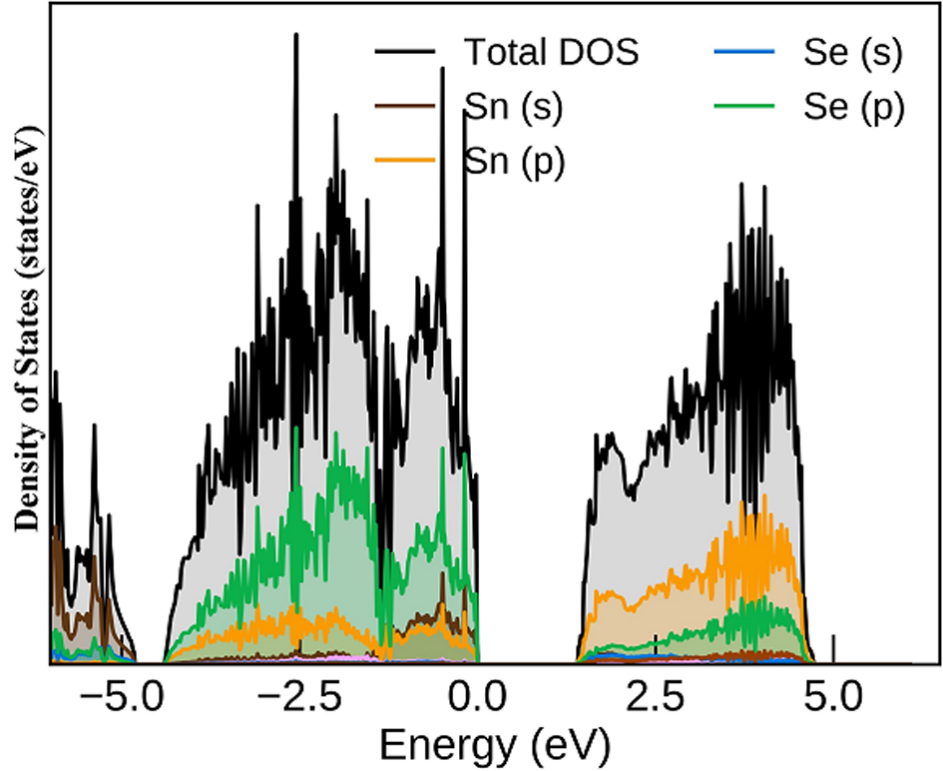
Density of states plot for the π-SnSe alloy calculated by the meta GGA-mBJ functional.

After optimizing the lattice structure of π-SnSe and obtaining the lattice parameter close to the experimental value, we investigated the electronic structure of π-SnSe. The density of states (DOS) plot for the π-SnSe calculated by the meta GGA-mBJ is presented in Figure 3. The upper valence band is majorly contributed by the p states of Se and Sn atoms with a small share of Sn s states, according to the revised lone pair model [58]. The conduction band of the π-SnSe phase consists of p states of Sn and Se as the majority of states and the share of s states of Sn and Se is very small, which can be seen from the projected DOS plot in Figure 3.

**Figure 4:**
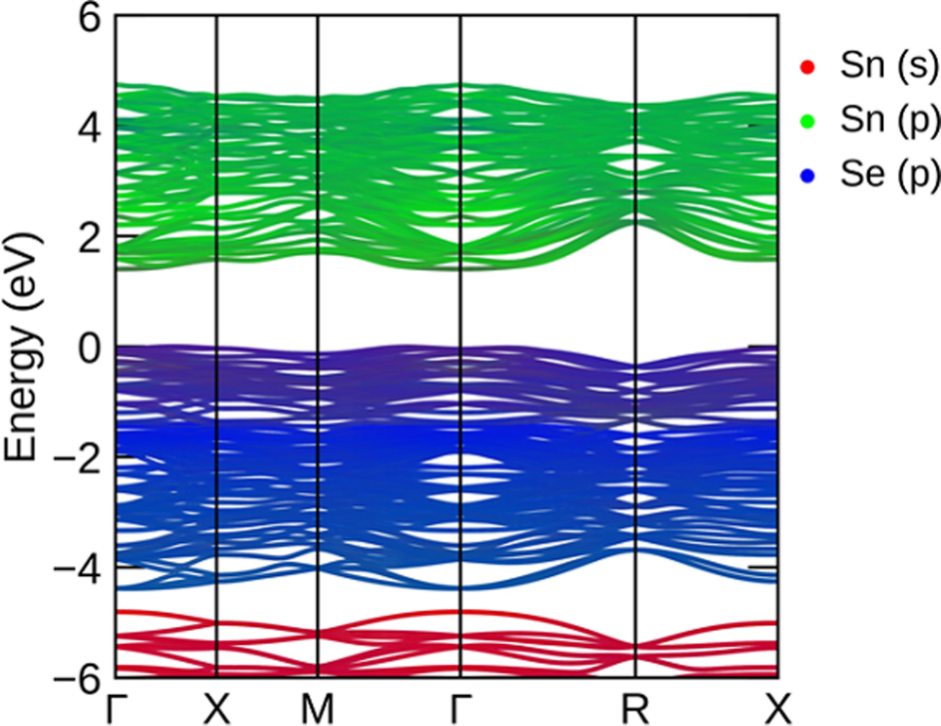
Projected band structure plot for the π-SnSe calculated by the meta GGA-mBJ functional.

The conventional electronic configuration of Sn [5s^2^5p^0^] and Se [4s^2^4p^4^] is theoretically helpful, yet the heteropolar bonding is nuanced. The projected electronic band structure of the cubic π-SnSe system is shown in Figure 4. The red, green, and blue colors represent the Sn s, Sn p, and Se p orbitals, respectively. The s states of Sn lie around the energy level of −5 to −6 eV. The valence bands before the Fermi level are mainly contributed by the p states of Sn and Se atoms whereas the p states of Se are mostly found in the energy range of −1.8–4.0 eV. The lower conduction band is mainly associated with p states of Sn. An indirect energy bandgap (*E*_BG_) of 1.078 eV was calculated by the PBE GGA, which is in good agreement (*E*_BG_ = 1.18 eV) with other DFT calculations [47], as it is a well-known fact that PBE-GGA underestimates the electronic bandgap values [59]. To calculate the bandgap value more accurately, we have also used the computationally inexpensive PBE-mBJ functional. For the cubic phase of π-SnSe, we obtained an indirect bandgap of 1.402 eV, along with the X to Γ high-symmetry point, calculated from the PBE-mBJ functional, which is slightly larger but quite consistent with the experimental bandgap value of 1.28 eV [47]. The bandgap of π-SnSe is larger than that of α-SnSe which has an indirect bandgap of 0.9 eV [1]. It can be also visualized that the band structure of the π-SnSe phase is distorted as compared to the band structure of the ideal rock-salt SnSe phase (64 atoms) which has a very small bandgap of 0.2 eV [47].

### Thermodynamic properties

It is very crucial to know thermodynamic (TD) properties to obtain more details about the particular responses of an alloy, especially when it is subjected to critical limitations, such as high-pressure and high-temperature conditions. Therefore, it is essential to examine the effects of temperature as well as pressure on TD parameters, such as the Grüneisen parameter (γ), Debye temperature (θ_D_), thermal expansion coefficient (α), heat capacity (*C*_V_), and volume. We employed the quasi-harmonic Debye model [60–61] to explore the TD properties of the π-SnSe alloy. We obtained the TD properties at some fixed pressure range of 0–40 GPa along with the temperature variation of 0–800 K.

**Figure 5:**
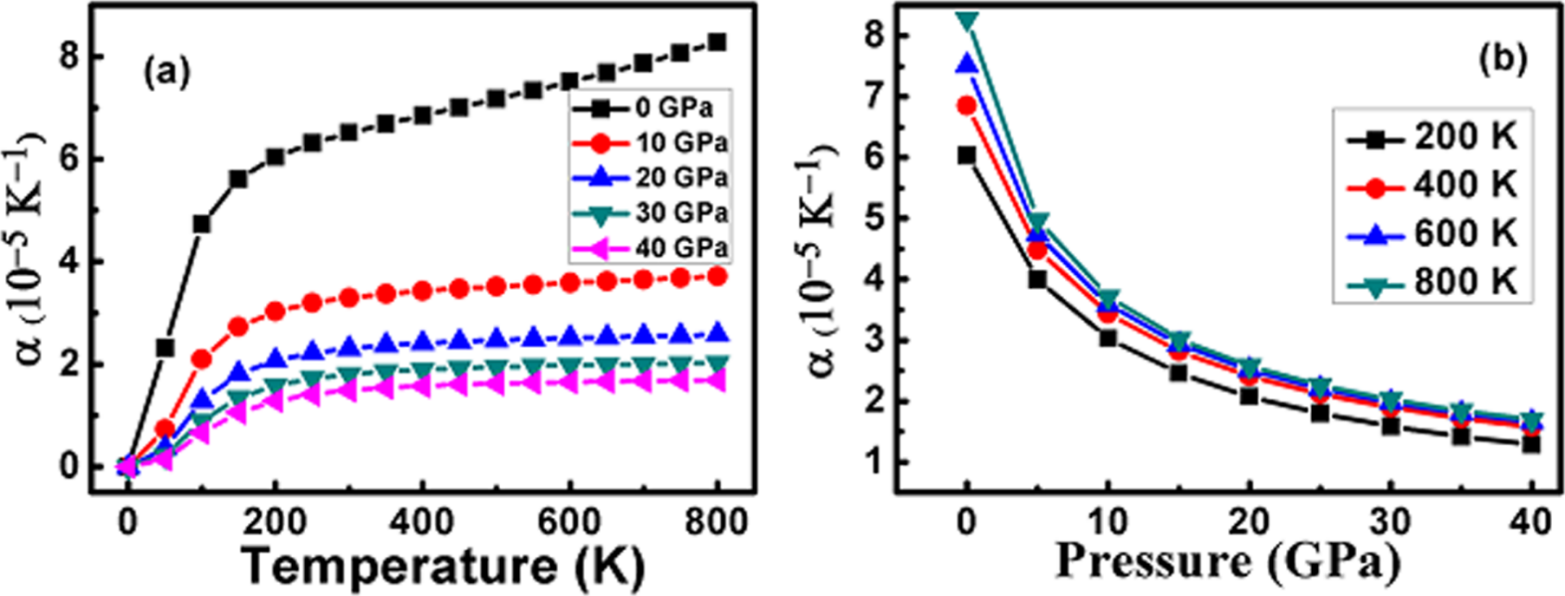
Variation of the thermal expansion coefficient as a function of (a) temperature and (b) pressure for the π-SnSe alloy.

The thermal expansion coefficient is an important parameter used to predict the TD equation of state and has experimental and theoretical significance. The temperature and pressure variation of the thermal expansion coefficient is presented in Figure 5a along with the pressure. The thermal expansion coefficient reveals the information about the amplitude regarding atomic lattice vibration which demonstrates how the alloy dimension changes when the external temperature is applied. It can be seen that by maintaining the pressure (temperature) fixed and by changing the temperature (pressure), respectively, a high increase (decrease) can be observed from Figure 5a and Figure 5b in the thermal expansion coefficient. At a specific temperature, the expansion coefficient decreases exponentially by the increase of pressure. For an applied pressure, a sharp increase can be seen in the thermal coefficient around room temperature. For temperature values higher than room temperature, α converges towards an approximately constant value. For our DFT calculations applied to the π-SnSe alloy, α is 6.51 × 10^−5^ K^−1^ at zero pressure and 300 K.

**Figure 6:**
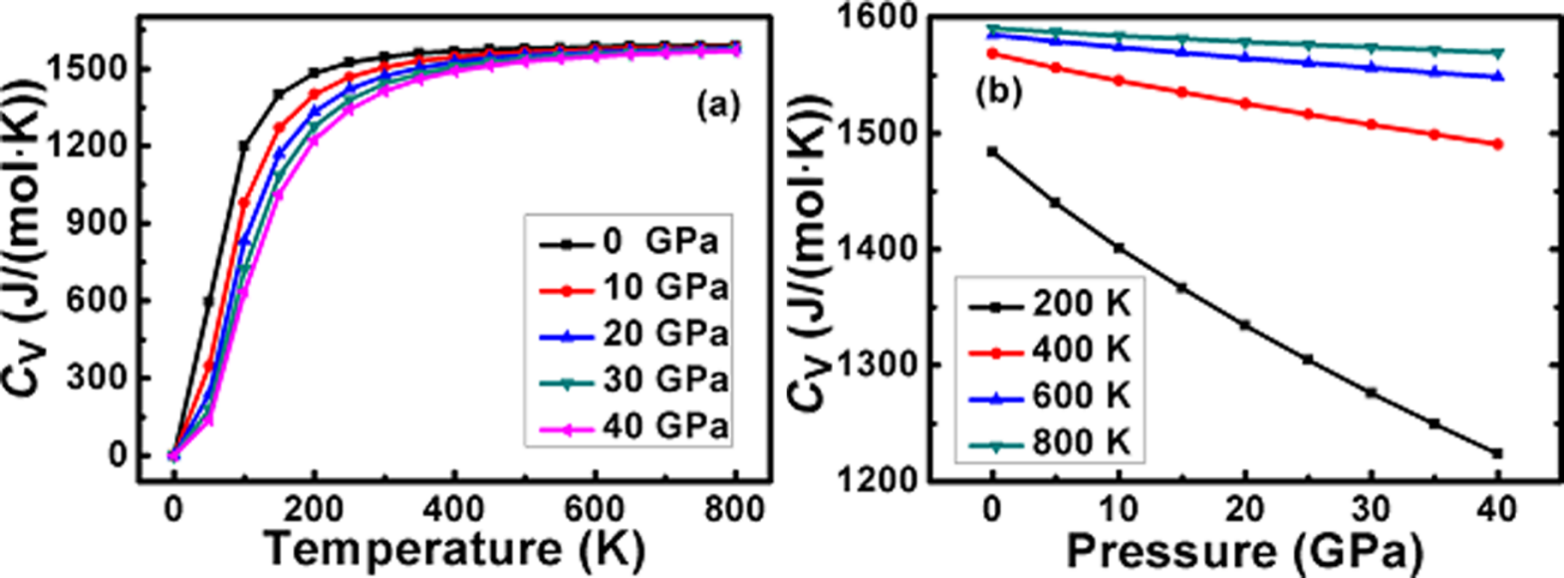
Variation of heat capacity as a function of (a) temperature and (b) pressure for the π-SnSe alloy.

The heat capacity value of an alloy can reveal its lattice vibrational properties. Thus, a variation of the heat capacity is calculated as a function of the temperature at some fixed pressure values for the π-SnSe alloy, as shown in Figure 6a and Figure 6b. It can be easily visualized from Figure 6a that for a fixed pressure, the *C*_V_ curve grows sharply up to 300 K then the value of *C*_V_ gradually raises. Afterward, at a higher temperature range, the *C*_V_ curve keeps a continuing pace and tends to reach the Dulong–Petit limit, revealing that at a high-temperature range all the phonon modes get excited by the thermal energy, which is a typical behavior for all solids at a high temperature [61–62]. It can be seen from Figure 6 that at a temperature higher than 300 K, *C*_V_ depends on the pressure as well as temperature and satisfy the relation *C*_V_ ∝ *T*^3^ [63]. From Figure 6 it can be also noted that the effects of temperature on *C*_V_ are more substantial as compared to the pressure. It can also be seen that temperature and pressure possess the opposite impact on the value of *C*_V_ for the π-SnSe alloy. At 800 K, the maximum value of the heat capacity 1590.26 J/(mol·K) is observed, whereas at 300 K (at 0 GPa), the measured value of heat capacity is 1546.09 J/(mol·K).

**Figure 7:**
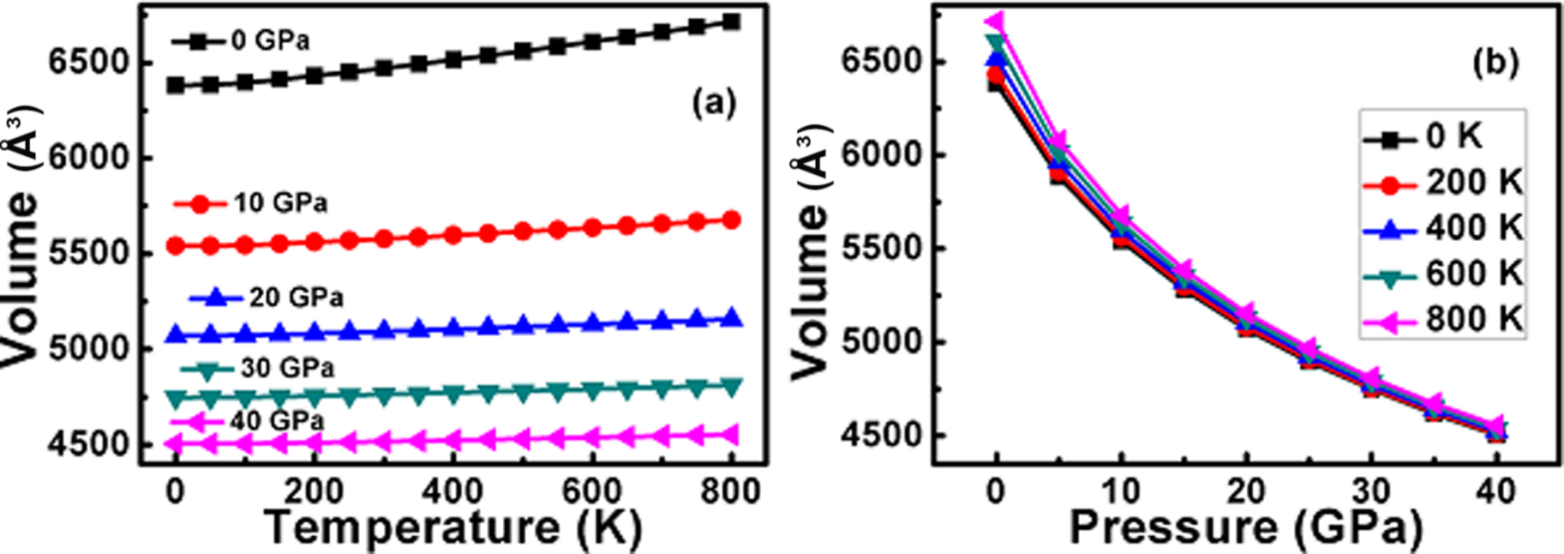
Variation of the primitive unit cell volume as a function of (a) temperature and (b) pressure for the π-SnSe alloy.

Figure 7 represents the variation of the primitive unit cell volume of the π-SnSe alloy as a function of the temperature or pressure. It is apparent from Figure 7a that upon a temperature increase, the volume steadily increases, and above 300 K the volume change is more significant. Yet, this particular pattern gets much less notable at a higher pressure range (i.e., 10–40 GPa). On the other hand (Figure 7b), the effect of pressure at a certain temperature represents a gradual decrease in volume with increasing pressure.

**Figure 8:**
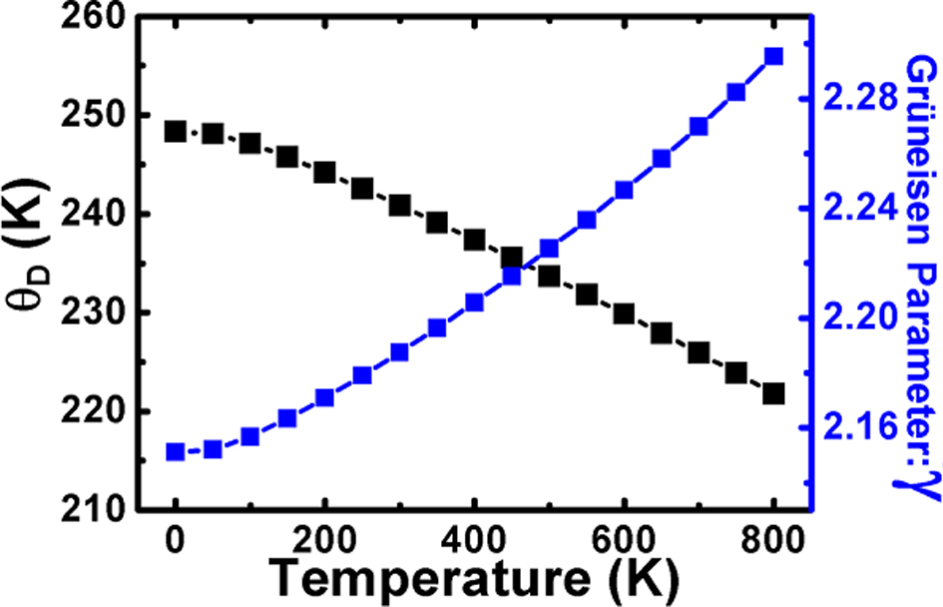
Variation of Debye temperature (θ_D_) and the Grüneisen parameter (γ) at different temperature values for the π-SnSe alloy.

Finally, the Grüneisen parameter (γ) and the Debye temperature (θ_D_) have been predicted with a temperature variation at zero pressure. These TD parameters have practical importance and are two of the most important thermodynamic parameters linked to the various material properties. The Grüneisen parameter shows the interaction between phonons and the lattice volume. It relates to the vibrational frequency change when the volume of the crystal lattice is varied. It also demonstrates the effect of temperature variations on the size or dimensions of the crystal structure. The Debye temperature can be defined as the highest temperature which can be reached due to individual vibration modes. Thus, these two important quantities (i.e., the Grüneisen parameter (γ) and the Debye temperature (θ_D_)) are calculated as a function of temperature at zero pressure and presented in Figure 8. It can be seen that the θ_D_ gradually decreases with temperature increase. For the π-SnSe alloys, the θ_D_ value calculated via DFT at room temperature and zero pressure is 240.86 K. On the other hand, γ linearly increases with temperature at zero pressure. The value of the Grüneisen parameter is 2.187 at zero pressure for the π-SnSe system.

So far, to the best of our knowledge, we did not find any theoretical or experimental results regarding TD properties of the π-SnSe alloy to compare with. Therefore, our DFT investigations will probably give a useful reference for additional research.

### Thermoelectric properties

To understand the thermoelectric (TE) response and applicability of the cubic π-SnSe alloy, we employed the semiclassical Boltzmann transport theory to determine the Seebeck coefficient (*S*), the electrical conductivity (σ/τ), as well as the electronic part of the thermal conductivity (κ_e_/τ) by applying the following relations [9]:

[3] $$\sigma_{\alpha \beta }\left(T\right)=\frac{1}{\Omega }\int \sigma_{\alpha \beta }\left(\varepsilon \right)\left[-\frac{\partial f_{0}\left(T,\varepsilon \right)}{\partial \varepsilon }\right]\text{d}\varepsilon ,$$

[4] $$\kappa_{\alpha \beta }^{0}\left(T\right)=\frac{1}{e^{2}T\Omega }\int \sigma_{\alpha \beta }\left(\varepsilon \right){\left(\varepsilon \right)}^{2}\left[-\frac{\partial f_{0}\left(T,\varepsilon \right)}{\partial \varepsilon }\right]\text{d}\varepsilon ,$$

[5] $$S_{\alpha \beta }\left(T\right)=\frac{1}{eT\Omega \sigma_{\alpha \beta }\left(T\right)}\int \sigma_{\alpha \beta }\left(\varepsilon \right)\left(\varepsilon \right)\left[-\frac{\partial f_{0}\left(T,\varepsilon \right)}{\partial \varepsilon }\right]\text{d}\varepsilon .$$

Here, *f*_0_(*T*,ε) is the Fermi distribution function, α and β are tensor indices, Ω is cell volume, and σ_αβ_(ε) is the electrical conductivity tensor computed through Fourier interpolation of the band energies. We have evaluated these TE parameters under a constant relaxation time (τ ≈ 10^−14^ s) within a temperature range of 0–1000 K.

**Figure 9:**
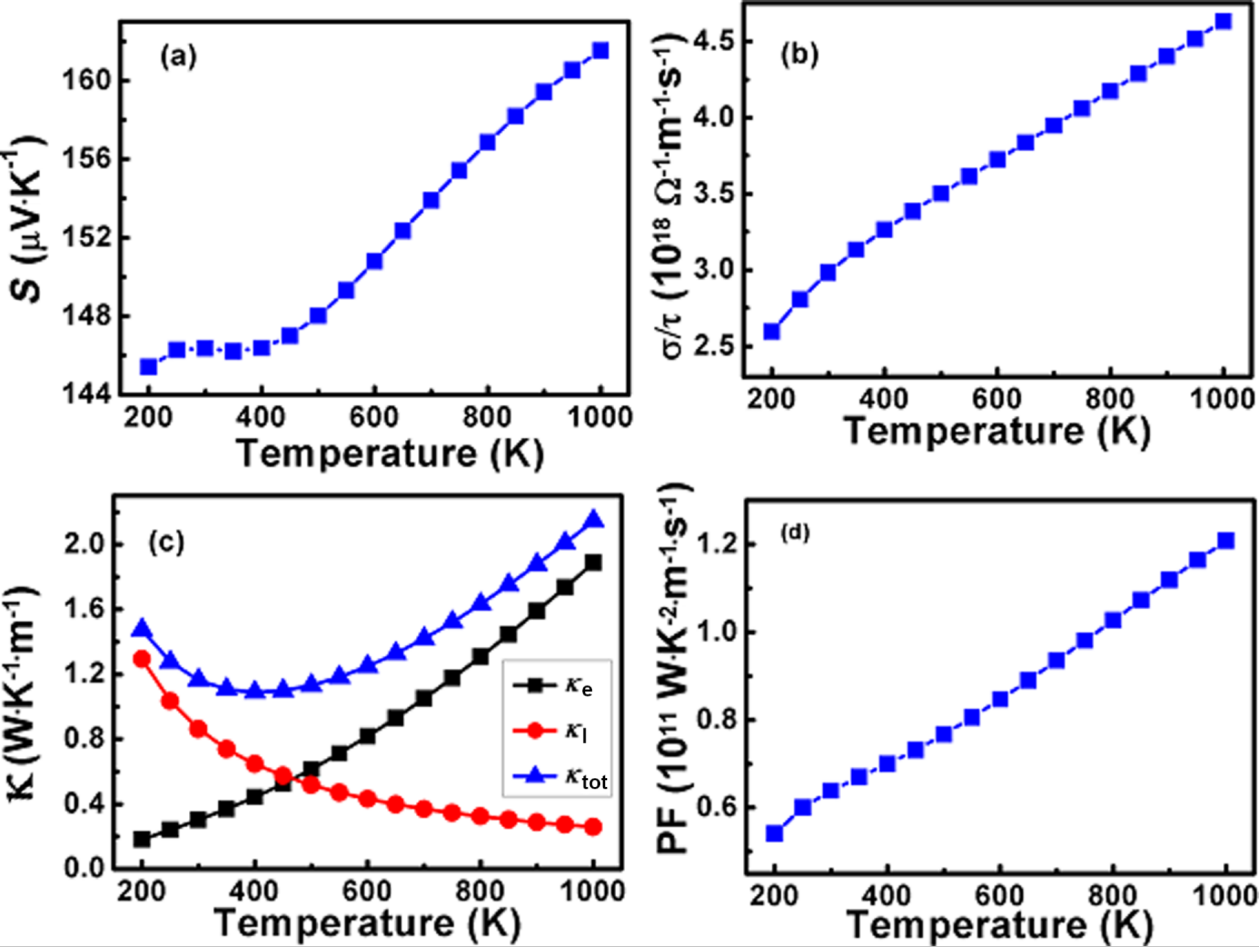
Temperature-dependent (a) Seebeck coefficient (*S*); (b) electrical conductivity (σ/τ); (c) electronic (κ_e_), lattice (κ_l_), and total (κ_tot_) thermal conductivity values and (d) power factor (PF) of the π-SnSe alloy.

The Seebeck coefficient is an important material property to evaluate, which can be described as the voltage produced per temperature unit and it shows that the electron carries both current and heat. It provides an intriguing correlation between the electronic structure and nature of the dominant charge carriers of an alloy. A high value of the Seebeck coefficient is needed for an excellent TE device. A variation of the Seebeck as a function of temperature is presented in Figure 9a. It can be observed that the Seebeck coefficient value at a lower temperature (200–400 K) remains almost the same ≈146 μV·K^−1^ and then linearly increases with an increasing temperature. The maximum value of the Seebeck coefficient of 161.52 μV·K^−1^ is obtained for the π-SnSe alloy at the highest temperature (1000 K). We obtained the positive value of the Seebeck coefficient for the studied π-SnSe alloy for all the considered temperature ranges, which suggests that holes are the main charge carriers and confirms the p-type conduction. Moreover, for the studied π-SnSe system, valence band maxima and conduction band minima are situated at low symmetry points (i.e., along the X and Γ directions, respectively), which means that the electronic properties of the studied π-SnSe alloy provide a high value of the Seebeck coefficient [64]. The high value of the Seebeck coefficient for the studied π-SnSe semiconductor alloy shows that it can be used for building excellent TE devices.

Another important TE parameter is the electrical conductivity, which is shown in Figure 9b for the cubic π-SnSe alloy as a function of temperature. The electrical conductivity parameter describes the flow of charge carriers from high- to low-temperature regions. We found that the electrical conductivity is 2.98 × 10^18^ Ω^−1^·m^−1^·s^−1^ at room temperature for the studied material. The electrical conductivity linearly increases with temperature increase, since at a high temperature range the concentration of charge carriers (holes) is largely due to the gained kinetic energy. The maximum electrical conductivity value of the π-SnSe alloy is 4.63 × 10^18^ Ω^−1^·m^−1^·s^−1^ at 1000 K.

In solids, the total thermal conductivity (κ_tot_) originates due to holes/electrons drifting throughout the crystals along with phonons and heat flow. It is the combination of the electronic part of the thermal conductivity (κ_e_) and the lattice thermal conductivity (κ_l_), which depends upon the number of carrier concentrations. The electronic part of the thermal conductivity, which is produced through the movement of the electrons across the temperature gradient, is presented in Figure 9c for the studied π-SnSe alloy. At the ambient condition, the value of (κ_e_) is observed to be 0.30 × 10^18^ W·K^−1^·m^−1^ for the cubic π-SnSe alloy under a constant relaxation time (10^14^). The thermal conductivity linearly increases with the increasing temperature which can be also seen from Figure 9c and has a relatively similar linear increase trend as that of the electrical conductivity. This specific outcome is in agreement with the Wiedemann–Franz law [65], confirming the proportional relationship between electrical and thermal conductivity. The maximum value of thermal conductivity of the π-SnSe alloy is 0.30 × 10^18^ W·K^−1^·m^−1^ at 1000 K. The lattice thermal conductivity (κ_l_) of the cubic π-SnSe alloy is estimated by using the Slack’s equation as the BoltzTraP code only calculates the electronic part of the thermal conductivity. The mathematical relation required to calculate the lattice thermal conductivity can be expressed as [66]:

[6] κl=AθD3Va13mavγ2n23T,

where

[7] $$A=\frac{2.43\times 10^{-6}}{1-\frac{0.514}{\gamma }+\frac{0.228}{\gamma^{2}}}.$$

In the above expressions, the *A* parameter is equivalent to ≈3.14 × 10^−8^, which is a dimensionless collection of physical constants. The Debye temperature is represented by θ_D_, *V*_a_ is the volume per atom, *m*_av_ is the average atomic mass, *n* is the total number of atoms present in the unit cell, and γ is the Grüneisen parameter [9]. We used the values of θ_D_ = 240.86 and γ = 2.1875 at 300 K in the Slack’s equation to obtain the lattice thermal conductivity. The temperature dependence of the lattice thermal conductivity for the cubic π-SnSe alloy is presented in Figure 9c. From the plot, it can be noted that lattice thermal conductivity decreases with an increase in temperature. At 300 K, the lattice thermal conductivity was 0.86 W·m^−1^·K^−1^ and it decreases to 0.86 W·m^−1^·K^−1^ at 1000 K. The decrease in the lattice thermal conductivity is mainly a result of lattice distortion generated through a large entropy impact which accelerates the phonon–phonon scattering process. The total thermal conductivity (κ_tot_) for the π-SnSe alloy is also plotted and presented in Figure 9c. It can be noted that initially, by the increase in temperature, κ_tot_ decreases and reaches its minimum value of 1.089 W·m^−1^·K^−1^ at 400 K. After 400 K, κ_tot_ gradually starts to linearly increase and has the highest value of 2.15 W·m^−1^·K^−1^ at 1000 K.

The power factor is also calculated to approximate the figure of merit for the studied π-SnSe alloy, which is presented in Figure 9d as a function of temperature. The PF has a collective impact and is directly linked with the Seebeck coefficient along with the electrical conductivity. It can be observed from the PF plot that PF starts to linearly increase with the temperature increase and has a similar increasing trend as that of the Seebeck coefficient and the electrical conductivity. The maximum PF value of 1.20 × 10^11^ W·K^−2^·m^−1^·s^−1^ is achieved at 1000 K, whereas PF has a value of 0.63 × 10^11^ W·K^−2^·m^−1^·s^−1^ at room temperature.

**Figure 10:**
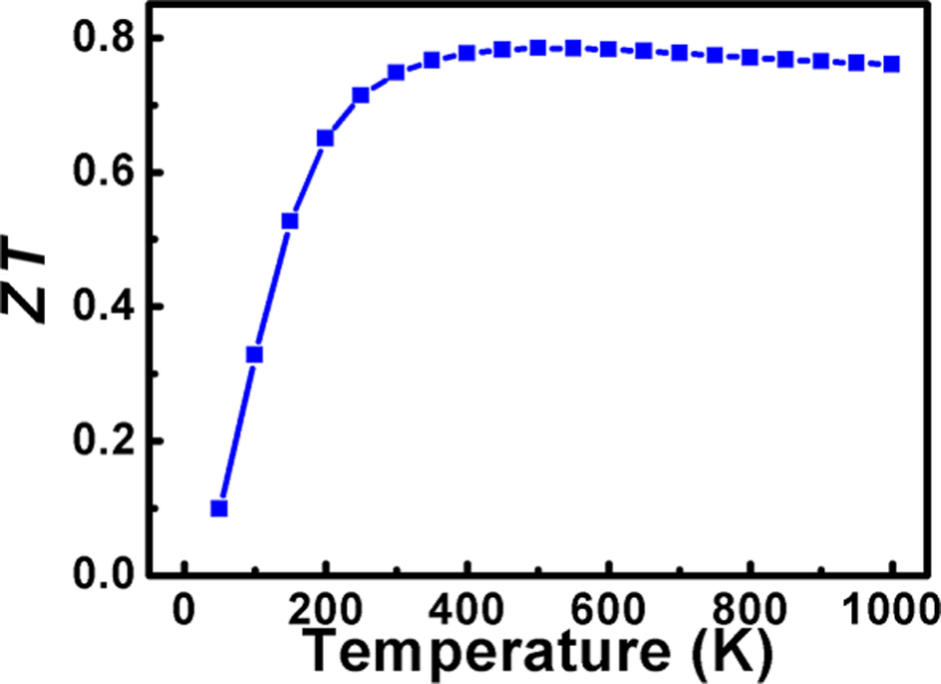
Figure of merit of the cubic π-SnSe as a function of temperature.

The figure of merit of π-SnSe investigated via our DFT method is presented in Figure 10. At room temperature, a *ZT* value of 0.74 is obtained which slightly increases with the temperature increase, reaching its maximum value of 0.76 at 1000 K. Our DFT-studied π-SnSe alloy has a quite high value of *ZT* ≈ 0.74 at low and high temperature values, which is very promising for thermoelectric applications. On the other hand, for the p-type α-SnSe alloy, a remarkably high value of *ZT* ≈ 2.6 at 773 K along the *b* axis has been reported, which is the world record so far [1,5]. Also, for the SnSe thin films and 2D sheets, the experimental *ZT* values were relatively low; the maximum value can only reach 0.28 at 675 K [67–68]. One recent study shows that the Gd-doped 2D SnSe material shows a *ZT* value of ≈1 at 868 K, which is high as compared to π-SnSe [69]. It is very important to mention that we evaluated the transport properties only under constant relaxation time approximation. For the practical use of the π-SnSe alloy in TE applications, further experimental studies are needed to calculate the *ZT* value with an accurate evaluation of the relaxation time.

### Optical properties

The optical properties in solid materials are governed by the response of the electrons to time-dependent electromagnetic perturbations triggered by the shining of light. As a result, the computation of the optical properties is decreased towards the assessment of the response function, which is called dielectric tensor or polarizability. For practical optoelectronics applications, it is essential to investigate the optical response of the π-SnSe alloy. For this purpose, we have used the independent particle approximation (IPA) implemented by the optics module of WIEN2k, which computes the direct transitions through Kohn and Sham (KS) [70] eigenvalues between occupied and unoccupied states [71]. For the first step, a complex dielectric function ε(ω), which depends on the frequency and comprises all the details of the optical response, is determined through the following relation [72]:

[8] $$\varepsilon \left(\omega \right)=\varepsilon_{1}\left(\omega \right)+\varepsilon_{2}\left(\omega \right),$$

where ε_1_(ω) and ε_2_(ω) represent the real and imaginary parts of the dielectric function, respectively. The absorptive imaginary ε_2_(ω) component can be determined by calculating the matrix entries of the momentum operator 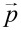 between the filled and unfilled energy states by applying the following relation [71]:

[9]
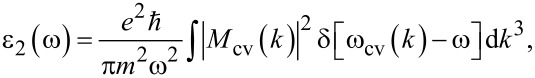


where the potential vector δ describes the electric field component, *k* is the reciprocal lattice vector, *M*_cv_(*k*) is equivalent to dipole moment values, v and c represent the valence and conduction bands, respectively. The relation *ħ*ω_cv_ = *E*_c_*_k_* – *E*_v_*_k_* shows the transition energy from valence to conduction bands. This absorptive imaginary ε_2_(ω) part of the dielectric function leads to the real part ε_1_(ω) through the Kramers–Kronig transformation and can be obtained from the following relation [73]:

[10] $$\varepsilon_{1}\left(\omega \right)=1+\frac{2P}{\pi }\int_{0}^{\infty }\frac{\varepsilon_{2}\left(\omega^{'}\right)\omega^{'}}{\omega^{'2}-\omega^{2}}\text{d}\omega^{'}.$$

where *P* and ω represent the principal value and angular frequency, respectively. The other optical parameters, such as the absorption coefficient α(ω), energy loss function *L*(ω), refractive index *n*(ω), extinction coefficient *K*(ω), reflectivity *R*(ω), and conductivity σ(ω) can be easily obtained from the dielectric function by using the following expressions [72]:

[11] $$\alpha \left(\omega \right)=2\omega \sqrt{\frac{{\left[\varepsilon_{1}^{2}\left(\omega \right)+\varepsilon_{2}^{2}\left(\omega \right)\right]}^{\frac{1}{2}}-\varepsilon_{1}\left(\omega \right)}{2},}$$

[12] L(ω)=−Im(1ε(ω)),

[13] $$n\left(\omega \right)=\sqrt{\frac{\left[\left|\varepsilon \left(\omega \right)\right|+\varepsilon_{1}\left(\omega \right)\right]}{2},}$$

[14] $$K\left(\omega \right)={\left(\frac{\sqrt{\varepsilon_{1}^{2}\left(\omega \right)+\varepsilon_{2}^{2}\left(\omega \right)-\varepsilon_{1}\left(\omega \right)}}{2}\right)}^{2},$$

[15] $$R\left(\omega \right)=\left|\frac{\sqrt{\varepsilon \left(\omega \right)}-1}{\sqrt{\varepsilon \left(\omega \right)}+1}\right|,$$

[16] σ(ω)=ω4πImε(ω).

**Figure 11:**
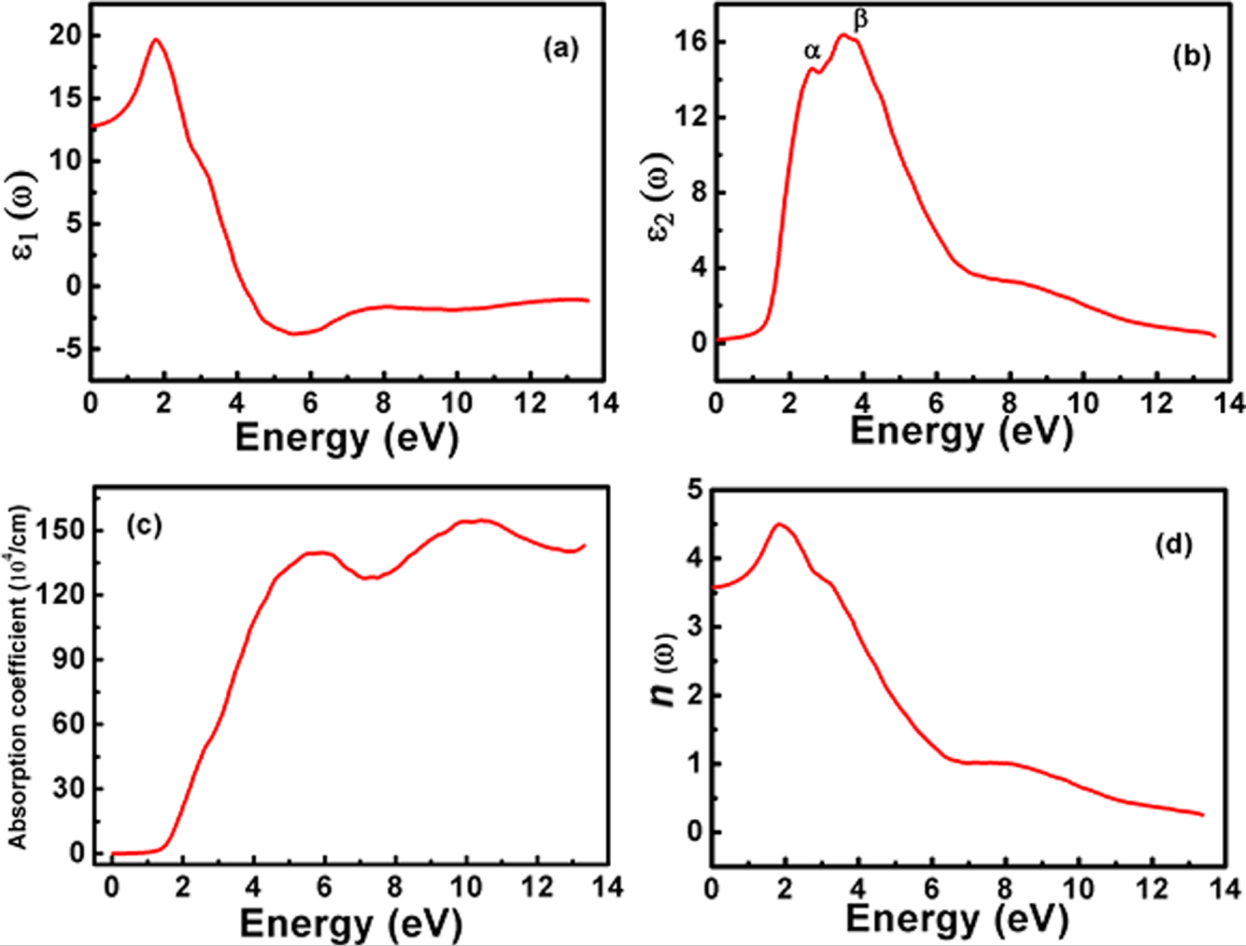
Variation of the (a) real ε_1_(ω) and (b) imaginary ε_2_(ω) parts of the dielectric function. (c) The absorption coefficient spectra α(ω) and (d) the refractive index *n*(ω) as a function of energy for the cubic π-SnSe alloy, computed within the independent particle approximation centered on single-particle electronic eigenvalues obtained from the Tb-mBJ functional.

The real part ε_1_(ω) of the dielectric tensor describes the polarization and the induced scattering of the incident light photons, while ε_2_(ω) refers to the magnitude of absorption of the incident radiations. The real part ε_1_(ω) of the cubic π-SnSe alloy is presented in Figure 11a as a function of the photon energy. It can be observed that for the π-SnSe system, the static dielectric constant ε_1_(0) is 12.82, which is positive. The positive value of the real dielectric tensor ε_1_(ω) suggests that the studied material is a semiconductor and transparent. There is only one peak that can be observed for the π-SnSe alloy in the visible energy range. Initially, by increasing the photon energy (*ħ*ω), the value of ε_1_(ω) starts to increase until it reaches its maximum peak value of 19.65 at 1.75 eV. Afterward, ε_1_(ω) starts to gradually decrease and reaches its minimum value of −3.80 at 5.5 eV. Beyond this photon energy value, the value of ε_1_(ω) shows an increasing trend but stays negative at high energies.

The imaginary part ε_2_(ω) of the dielectric tensor of the studied π-SnSe system as a function of photon energy is presented in Figure 11b, which is directly linked to the electronic properties (i.e., band structure and DOS). This absorption of photons accounts for interband transitions within semiconductors from a certain location of the electromagnetic spectrum. Most transitions happen within the infrared region once the energy is under 0.1 eV. Two unique absorption peaks of ε_2_(ω) can be observed from Figure 11b, which are marked as α and β for the π-SnSe alloy. It can be noticed that for the π-SnSe alloy, the ε_2_(ω) threshold point is equivalent to its energy bandgap which determines the energy extent to stimulate electron transitions. The maximum values of the peaks α and β are 14.54 and 16.36 at the photon energy of 2.73 and 3.55 eV, respectively. Theoretically, the possible transitions can be identified from the ε_2_(ω) spectra. For the π-SnSe, DOS, and band structure plots (Figure 3 and Figure 4), the first transition peak α can be generated from the Se p orbital in the valence band to the Sn p orbital in the conduction band. The second peak β is less intense as compared to the α peak, which can be due to the transition from Sn s to Se p orbitals as it can be seen from the DOS plot (Figure 3). However, it can be noticed that two important peaks (α and β) are located in the visible region. Beyond the β peak, ε_2_(ω) shows a decreasing trend at higher energies.

The absorption coefficient α(ω) is presented in Figure 11c as a function of photon energy. The absorption coefficient demonstrates all the information of the entire optical region of an alloy and shows the absorption peaks from the occupied to unoccupied states which occur due to electronic transitions. The studied π-SnSe alloy reveals the broader part of the electromagnetic spectrum. It can be noted from the absorption spectra (Figure 11c) that the value of α(ω) π-SnSe is zero until the photon energy is lower as compared to its energy bandgap of 1.41 eV. After this energy, the π-SnSe alloy displays a sharp increase of absorption coefficient α(ω) up to 139.6 10^4^/cm at 6 eV. After this energy value, α(ω) starts to decrease with the increase in photon energy, and then another broader peak is observed at ≈10 eV in which α(ω) reaches its maximum value of 154.23 10^4^/cm. From the literature, we have noted that the α-SnSe has an indirect bandgap of 0.9 eV and a direct gap of 1.3 eV [5,74–76]. On the other hand, the optical bandgap value of the 2D SnSe is 1.10 eV [77]. In the present study, our DFT computed results for the π-SnSe shows that it has an indirect bandgap of 1.405 eV and a direct gap of 1.439 eV. Therefore, π-SnSe has a considerably larger bandgap of ≈0.5 eV as compared to α-SnSe. It means that α-SnSe is operating in the short-wave infrared range. Alternatively, the π-SnSe is expected to be effective closer to the visible range, with technological implications in solar cells, thermoelectric applications, and photocatalysis [78]. The broader region along with the high values of the absorption coefficient for the π-SnSe indicates that this alloy can be used to yield exceptional solar and optical applications.

From the point of view of optical applications, the knowledge of refractive index *n*(ω) for any material is important. The refractive index *n*(ω) plot for the π-SnSe alloy is demonstrated in Figure 11d. The static refractive index *n*(0) is 3.58 for the π-SnSe. There is one important peak in the visible region and the maximum value of the refractive index *n*(ω) is observed to be 4.49 at 1.86 eV. Beyond this energy, the refractive index starts to rapidly decrease throughout the entire energy spectrum, but it remains positive.

**Figure 12:**
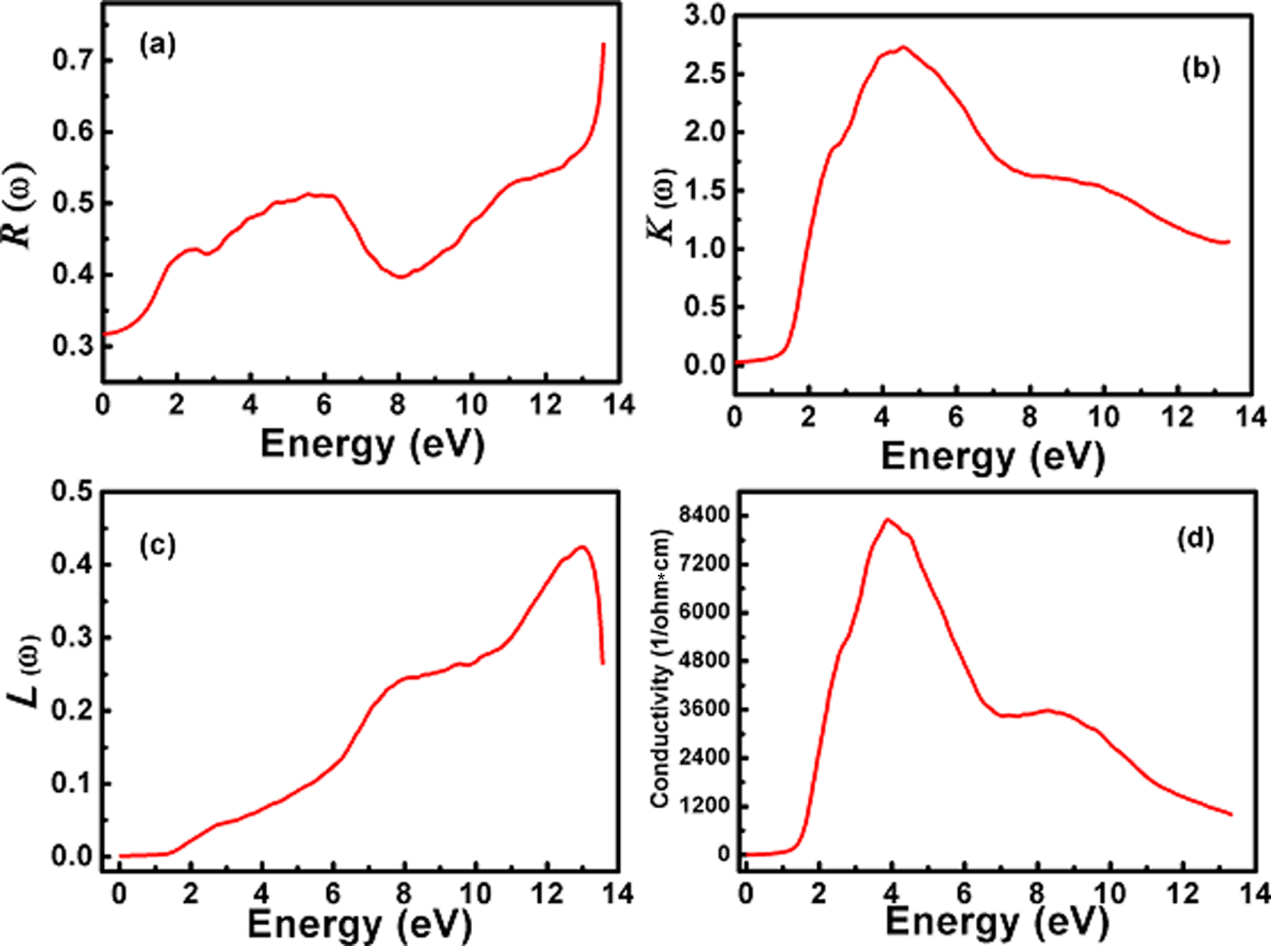
Calculated (a) reflectivity *R*(ω), (b) extinction coefficient *K*(ω), (c) energy loss function and (d) real optical conductivity as a function of energy for the cubic π-SnSe alloy. The extinction coefficient is illustrated in (b) as a function of photon energy. The static value of *K*(0) for the π-SnSe is negligibly small until it reaches its bandgap value (1.41 eV). After this energy value, the value of *K*(ω) starts to rapidly increase until it reaches its maximum value of 2.72 at 4.61 eV.

The reflectivity *R*(ω) of the material can be described as the change in the incident and reflected power. The reflectivity *R*(ω) of the π-SnSe alloy is also calculated and illustrated in Figure 12a. The static reflectivity *R*(0) for the π-SnSe is 31.74%. By increasing the incident photon energy, the reflectivity *R*(ω) increases. Around 6 eV, *R*(ω) decreases into a dip and then again shows an increasing trend over the high energy regime and reaches its maximum value of 72% at 13.5 eV. It is essential to mention that the π-SnSe alloy has the robust reflection at its surface as there is a strong reflection at the same energy range at which ε_1_(ω) has negative values.

Another very useful optical parameter is the energy loss function *L*(ω) which is also calculated to get information from the π-SnSe alloy when it is exposed to light (photons). The energy loss function explores the propagation of photon energies within tangled medium/alloy at a photon energy greater than its bandgap value. Figure 12c illustrates the electron energy loss as a function of photon energy for the π-SnSe alloy. As it can be seen from the *L*(ω) plot, the propagation of electron energy loss starts to gradually appear at a photon energy greater than its bandgap value. The electron energy loss curve achieves its peak value around 13 eV and then begins to abruptly fall until it reaches 13.5 eV. This observed highest peak of *L*(ω) represents the plasma resonance of the π-SnSe alloy and their correlated frequencies, called plasma frequencies [79]. No dispersion or scattering is observed in *L*(ω) as the curve is linear between 0 and 1.4 eV.

The real optical conductivity σ(ω) is a valuable tool to evaluate the concentration of electrons that participate in optical transitions. The optical conductivity plot for the π-SnSe alloy is presented in Figure 12d as a function of photon energy. It can be seen from Figure 12d that there is no real optical conductivity σ(ω) found in the optical bandgap of this compound, which proves the nonexistence of electrons in unoccupied bands. After the photon energy value of 1.4 eV, there is an abrupt increase trend of the optical conductivity which directs the electronic transitions from valence to conduction bands. Only one peak is observed at the 4.2 eV and σ(ω) starts to linearly decrease at the entire higher energy regime.

## Conclusion

A detailed study has been performed on the structural, optoelectronic, thermodynamic, and thermoelectric properties of the π-SnSe alloy by first-principles calculations. The π-SnSe has a cubic phase (12.2 Å, *P*2_1_3), belongs to a class of non-centrosymmetric crystals which comprises 64 atoms per unit cell. Our DFT electronic calculations reveal that π-SnSe has an indirect bandgap (1.41 eV), is a p*-*type semiconductor, and a possible alternative to orthorhombic α-SnSe for thermoelectric applications at room and high temperatures. The mechanical stability is confirmed for the α-SnSe alloy as all the calculated mechanical parameters show a linear increasing trend by increasing pressure. From the Slack’s equation, we found an exceptionally low lattice thermal conductivity of 0.25 W·m^−1^·K^−1^ at 1000 K. For the studied material, a *ZT* value of 0.74 is achieved at room temperature. A linear behavior is observed with the increase of temperature for the transport parameters, such as the Seebeck coefficient, electrical and electronic conductivity, and power factor. At room temperature, the Seebeck coefficient and power factor have values of 146.37 µV·K^−1^ and 0.63 ×10^11^ W·K^−2^·m^−1^·s^−1^, respectively. The π-SnSe phase features a strong optical absorption onset and an intraband transition between Se p to Sn p orbitals at the near-infrared region, making it promising for applications in solar energy conversion. This work provides a promising platform for performing experimental work on thermoelectric applications of π-SnSe. Besides, the remarkable thermoelectric and optoelectronic properties of the π-SnSe can be regulated by using suitable doping and structural transformations.
